# Phylogenetic Analysis of Phenotypically Characterized *Cryptococcus laurentii* Isolates Reveals High Frequency of Cryptic Species

**DOI:** 10.1371/journal.pone.0108633

**Published:** 2014-09-24

**Authors:** Kennio Ferreira-Paim, Thatiana Bragine Ferreira, Leonardo Andrade-Silva, Delio Jose Mora, Deborah J. Springer, Joseph Heitman, Fernanda Machado Fonseca, Dulcilena Matos, Márcia Souza Carvalho Melhem, Mario León Silva-Vergara

**Affiliations:** 1 Department of Infectious and Parasitic Diseases, Triangulo Mineiro Federal University, Uberaba, Minas Gerais, Brazil; 2 Department of Molecular Genetics and Microbiology, Duke University Medical Center, Durham, North Carolina, United States of America; 3 Department of Medicine, Duke University Medical Center, Durham, North Carolina, United States of America; 4 Department of Pharmacology and Cancer Biology, Duke University Medical Center, Durham, North Carolina, United States of America; 5 Public Health Reference Laboratory, Adolfo Lutz Institute, São Paulo, São Paulo, Brazil; V.P.Chest Institute, India

## Abstract

**Background:**

Although *Cryptococcus laurentii* has been considered saprophytic and its taxonomy is still being described, several cases of human infections have already reported. This study aimed to evaluate molecular aspects of *C. laurentii* isolates from Brazil, Botswana, Canada, and the United States.

**Methods:**

In this study, 100 phenotypically identified *C. laurentii* isolates were evaluated by sequencing the 18S nuclear ribosomal small subunit rRNA gene (18S-SSU), D1/D2 region of 28S nuclear ribosomal large subunit rRNA gene (28S-LSU), and the internal transcribed spacer (ITS) of the ribosomal region.

**Results:**

BLAST searches using 550-bp, 650-bp, and 550-bp sequenced amplicons obtained from the 18S-SSU, 28S-LSU, and the ITS region led to the identification of 75 *C. laurentii* strains that shared 99–100% identity with *C. laurentii* CBS 139. A total of nine isolates shared 99% identity with both *Bullera* sp. VY-68 and *C. laurentii* RY1. One isolate shared 99% identity with *Cryptococcus rajasthanensis* CBS 10406, and eight isolates shared 100% identity with *Cryptococcus* sp. APSS 862 according to the 28S-LSU and ITS regions and designated as *Cryptococcus aspenensis* sp. nov. (CBS 13867). While 16 isolates shared 99% identity with *Cryptococcus flavescens* CBS 942 according to the 18S-SSU sequence, only six were confirmed using the 28S-LSU and ITS region sequences. The remaining 10 shared 99% identity with *Cryptococcus terrestris* CBS 10810, which was recently described in Brazil. Through concatenated sequence analyses, seven sequence types in *C. laurentii*, three in *C. flavescens*, one in *C. terrestris*, and one in the *C. aspenensis* sp. nov. were identified.

**Conclusions:**

Sequencing permitted the characterization of 75% of the environmental *C. laurentii* isolates from different geographical areas and the identification of seven haplotypes of this species. Among sequenced regions, the increased variability of the ITS region in comparison to the 18S-SSU and 28S-LSU regions reinforces its applicability as a DNA barcode.

## Introduction

The *Cryptococcus* genus includes more than 100 species of which most are considered non-pathogenic, with the exceptions of *Cryptococcus neoformans* and *Cryptococcus gattii*. During the last decade *Cryptococcus laurentii* has occasionally been described to infect severely immunocompromised hosts [1]–[3]. In most of these reports from which isolates were obtained, the blood and the cerebrospinal fluid (CSF) were the predominant sources [2]–[5].

*C. laurentii* was first identified from palm wine in the Congo by Kufferath in 1920 as *Torula laurentii* [6]. This isolate was then reclassified as *Torulopsis laurentii* [7] and renamed in 1950 as *Cryptococcus laurentii* (CBS 139) [8]. Later in Japan, an isolate with identical phenotypic characteristics was described as *Torula flavescens* [9], reclassified in 1922 as *Torulopsis flavescens* [7], and then renamed as *Cryptococcus flavescens* (CBS 942) [8].

*Cryptococcus aureus*, *Cryptococcus carnescens*, and *Cryptococcus peneaus*, in addition to *C. flavescens*, were also considered to be synonymous of *C. laurentii* until phylogenetic analysis of the internal transcribed spacer (ITS) and D1/D2 region of 28S nuclear ribosomal large subunit rRNA gene (28S-LSU) demonstrated that they are different species [10], [11].

In 2005, *Cryptococcus rajasthanensis* (CBS 10406) was described and differentiated from *C. laurentii* due to 1.6% and 7.5% divergence of the nucleotide identity of the 28-LSU and ITS regions, respectively [12]. More recently, *Cryptococcus terrestris* (CBS 10810), the cryptic species of *C. flavescens*, was isolated and described from soil in Brazil [13].

Currently, most *C. laurentii* isolates described around the world have been identified by morphological criteria, which can miss subtle differences and misidentify cryptic species [1], [14], [15]. Unlike *C. neoformans* and *C. gattii*, few studies have applied DNA sequencing to describe the molecular phylogeny of *C. laurentii* [11], [16], [17]. Thus, considering the potential pathogenicity of this species, this study aimed to evaluate the molecular phylogeny of clinical and environmental *C. laurentii* isolates through the sequencing of multiple ribosomal DNA regions.

## Methods

### Identification and fungal strains

We evaluated 100 environmental isolates of *C. laurentii* that were identified by classical mycological methods, such as India ink test, urease and phenoloxidase activity, thermotolerance at 37°C on Sabouraud dextrose agar, nitrate and carbon assimilation assays, carbohydrate fermentation, and microculture on cornmeal with Tween 80 [18], [19]. Of the 56 Brazilian isolates, 26 were obtained from peri-hospital areas, 5 from unidentified trees species, 7 from captive bird droppings in Uberaba, Minas Gerais State, and 18 from various environmental sources (bird droppings, trees, and air samples) from São Paulo State. The 18 isolates from Botswana were isolated from Mopane trees (*Colophospermum mopane*), the 14 isolates from New York State from Norway spruce (*Picea abies*) and trembling aspen (*Populus tremuloides*), and the 12 isolates from Vancouver, Canada from Douglas fir (*Pseudotsuga menziesii*) and other unidentified trees (Table 1). Isolates from Canada and the United States were isolated from swab samples collected in 2010 using single-swab BD CultureSwab Liquid Amies (Becton, Dickinson and Company, Sparks, Maryland, USA). The swabs were streaked onto yeast peptone dextrose agar (YPD, Becton, Dickinson and Company, Sparks, Maryland, USA) or Niger seed (NGS) agar containing chloramphenicol (0.5 g/L, Sigma-Aldrich, St. Louis, MO, USA), and yeast colonies were selected and colony purified [20].

**Table 1:** Isolate, species, source, and GenBank accession numbers of *Cryptococcus* spp. environmental isolates.

Isolate	Species	Source	Country	GenBank			Hap
				18S-SSU	28S-LSU	ITS	
CL01	*C. laurentii*	Peri-hospital	Brazil	JX393937	JN626983	JQ968462	1
CL02	*C. laurentii*	Peri-hospital	Brazil	JX393938	JN626984	JQ968463	1
CL03	*C. laurentii*	Peri-hospital	Brazil	JX393939	JN626985	JQ968464	1
CL04	*C. laurentii*	Peri-hospital	Brazil	JX393940	JN626986	JQ968465	1
CL05	*C. laurentii*	Peri-hospital	Brazil	JX393941	JN626987	JQ968466	1
CL06	*C. laurentii*	Peri-hospital	Brazil	JX393942	JN626988	JQ968467	1
CL07	*C. laurentii*	Peri-hospital	Brazil	JX393943	JN626989	JQ968468	1
CL08	*C. laurentii*	Peri-hospital	Brazil	JX393944	JN626990	JQ968469	1
CL09	*C. laurentii*	Peri-hospital	Brazil	JX393945	JN626991	JQ968470	1
CL10	*C. laurentii*	Peri-hospital	Brazil	JX393946	JN626992	JQ968471	1
CL11	*C. laurentii*	Trees	Brazil	JX393947	JN626993	JQ968472	1
CL12	*C. laurentii*	Peri-hospital	Brazil	JX393948	JN626994	JQ968473	1
CL13	*C. laurentii*	Peri-hospital	Brazil	JX393949	JN626995	JQ968474	1
CL14	*C. laurentii*	Peri-hospital	Brazil	JX393950	JN626996	JQ968475	1
CL15	*C. laurentii*	Peri-hospital	Brazil	JX393951	JN626997	JQ968476	1
CL16	*C. laurentii*	Peri-hospital	Brazil	JX393952	JN626998	JQ968477	1
CL17	*C. laurentii*	Peri-hospital	Brazil	JX393953	JN626999	JQ968478	1
CL18	*C. laurentii*	Trees	Brazil	JX393954	JN627000	JQ968479	1
CL19	*C. laurentii*	Peri-hospital	Brazil	JX393955	JN627001	JQ968480	1
CL20	*C. laurentii*	Peri-hospital	Brazil	JX393956	JN627002	JQ968481	1
CL21	*C. laurentii*	Peri-hospital	Brazil	JX393957	JN627003	JQ968482	1
CL22	*C. laurentii*	Trees	Brazil	JX393958	JN627004	JQ968483	1
CL23	*C. laurentii*	Peri-hospital	Brazil	JX393959	JN627005	JQ968484	1
CL24	*C. laurentii*	Peri-hospital	Brazil	JX393960	JN627006	JQ968485	1
CL25	*C. laurentii*	Trees	Brazil	JX393961	JN627007	JQ968486	1
CL26	*C. laurentii*	Peri-hospital	Brazil	JX393962	JN627008	JQ968487	2
CL27	*C. laurentii*	Peri-hospital	Brazil	JX393963	JN627009	JQ968488	1
CL28	*C. laurentii*	Peri-hospital	Brazil	JX393964	JN627010	JQ968489	1
CL29	*C. laurentii*	Peri-hospital	Brazil	JX393965	JN627011	JQ968490	1
CL30	*C. laurentii*	Peri-hospital	Brazil	JX393966	JN627012	JQ968491	1
CL32	*C. laurentii*	Peri-hospital	Brazil	JX393967	JN627013	JQ968492	1
CL33	*C. laurentii*	Pets shops	Brazil	JX393968	JN627014	JQ968493	1
CL34	*C. laurentii*	Pets shops	Brazil	JX393969	JN627015	JQ968494	1
CL35	*C. laurentii*	Pets shops	Brazil	JX393970	JN627016	JQ968495	1
CL36	*C. laurentii*	Pets shops	Brazil	JX393971	JN627017	JQ968496	1
CL37	*C. laurentii*	Pets shops	Brazil	JX393972	JN627018	JQ968497	1
CL38	*C. laurentii*	Pets shops	Brazil	JX393973	JN627019	JQ968498	1
CL39	*C. laurentii*	Pets shops	Brazil	JX393974	JN627020	JQ968499	1
E4	*C. laurentii*	Pigeon dropping	Brazil	JX393977	JX393999	JQ968502	3
E5	*C. laurentii*	Pigeon dropping	Brazil	JX393978	JX394000	JQ968503	3
E6	*C. laurentii*	Pigeon dropping	Brazil	JX393979	JX394001	JQ968504	3
E7	*C. laurentii*	Pigeon dropping	Brazil	JX393980	JX394002	JQ968505	1
E11	*C. laurentii*	Pigeon dropping	Brazil	JX393981	JX394003	JQ968506	3
E12	*C. laurentii*	Pigeon dropping	Brazil	JX393982	JX394004	JQ968507	3
E14	*C. laurentii*	Pigeon dropping	Brazil	JX393983	JX394005	JQ968508	3
DS288	*C. laurentii*	Mopane tree	Botswana	KC469712	KC485478	KC469756	1
DS386	*C. laurentii*	Mopane tree	Botswana	KC469715	KC485481	KC469759	1
DS388	*C. laurentii*	Mopane tree	Botswana	KC469716	KC485482	KC469760	1
DS390	*C. laurentii*	Mopane tree	Botswana	KC469717	KC485483	KC469761	1
DS392	*C. laurentii*	Mopane tree	Botswana	KC469718	KC485484	KC469762	1
DS394	*C. laurentii*	Mopane tree	Botswana	KC469719	KC485485	KC469763	4
DS400	*C. laurentii*	Mopane tree	Botswana	KC469720	KC485486	KC469764	5
DS402	*C. laurentii*	Mopane tree	Botswana	KC469722	KC485488	KC469766	6
DS403	*C. laurentii*	Mopane tree	Botswana	KC469723	KC485489	KC469767	5
DS444	*C. laurentii*	Mopane tree	Botswana	KC469724	KC485490	KC469768	6
DS447	*C. laurentii*	Mopane tree	Botswana	KC469726	KC485492	KC469770	4
DS455	*C. laurentii*	Mopane tree	Botswana	KC469727	KC485493	KC469771	1
DS529	*C. laurentii*	Norway spruce	USA	KC469728	KC485494	KC469772	3
DS530	*C. laurentii*	Norway spruce	USA	KC469729	KC485495	KC469773	3
DS531	*C. laurentii*	Norway spruce	USA	KC469730	KC485496	KC469774	3
DS619	*C. laurentii*	Norway spruce	USA	KC469735	KC485501	KC469779	3
DS620	*C. laurentii*	Norway spruce	USA	KC469736	KC485502	KC469780	3
DS621	*C. laurentii*	Norway spruce	USA	KC469737	KC485503	KC469781	3
DS744	*C. laurentii*	Douglas Fir tree	Canada	KC469742	KC485508	KC469786	3
DS746	*C. laurentii*	Douglas Fir tree	Canada	KC469743	KC485509	KC469787	3
DS748	*C. laurentii*	Douglas Fir tree	Canada	KC469744	KC485510	KC469788	3
DS778	*C. laurentii*	Tree	Canada	KC469745	KC485511	KC469789	5
DS782	*C. laurentii*	Tree	Canada	KC469746	KC485512	KC469790	5
DS783	*C. laurentii*	Tree	Canada	KC469747	KC485513	KC469791	5
DS784	*C. laurentii*	Tree	Canada	KC469748	KC485514	KC469792	5
DS785	*C. laurentii*	Tree	Canada	KC469749	KC485515	KC469793	5
DS797	*C. laurentii*	Tree	Canada	KC469750	KC485516	KC469794	3
DS798	*C. laurentii*	Tree	Canada	KC469751	KC485517	KC469795	3
DS802	*C. laurentii*	Tree	Canada	KC469752	KC485518	KC469796	3
DS806	*C. laurentii*	Tree	Canada	KC469753	KC485519	KC469797	5
CBS 139^T^	*C. laurentii*	Palm wine	Congo	AB032640	AF075469	AB035043	7
P482A	*C. rajasthanensis*	Tree	Brazil	JX393990	JX394017	JQ968514	8
CBS 10406^T^	*C. rajasthanensis*	Flowers	India	NA	AM262324	AM262325	NA
DS569	*C. aspenensis*	Trembling aspen	USA	KC469731	KC485497	KC469775	9
DS570	*C. aspenensis*	Trembling aspen	USA	KC469732	KC485498	KC469776	9
DS572	*C. aspenensis*	Trembling aspen	USA	KC469733	KC485499	KC469777	9
DS573^T^	*C. aspenensis* #	Trembling aspen	USA	KC469734	KC485500	KC469778	9
DS712	*C. aspenensis*	Trembling aspen	USA	KC469738	KC485504	KC469782	9
DS713	*C. aspenensis*	Trembling aspen	USA	KC469739	KC485505	KC469783	9
DS715	*C. aspenensis*	Trembling aspen	USA	KC469740	KC485506	KC469784	9
DS716	*C. aspenensis*	Trembling aspen	USA	KC469741	KC485507	KC469785	9
O242A	*C. flavescens*	Tree	Brazil	JX393984	JX394006	JQ968509	10
I113A	*C. flavescens*	Air	Brazil	JX393985	JX394007	JQ968510	11
I332A	*C. flavescens*	Tree	Brazil	JX393986	JX394008	JQ968511	10
I382A	*C. flavescens*	Tree	Brazil	JX393987	JX394009	KC469798	11
I243A	*C. flavescens*	Air	Brazil	JX393988	JX394010	JQ968512	11
I283A	*C. flavescens*	Air	Brazil	JX393989	JX394011	JQ968513	12
CBS 942^T^	*C. flavescens*	Air	Japan	AB085796	AB035042	AB035046	12
I572B	*C. terrestris*	Tree	Brazil	JX393991	JX394012	JQ968515	13
I573B	*C. terrestris*	Tree	Brazil	JX393992	JX394013	JQ968516	13
1B2011	*C. terrestris*	Pigeon dropping	Brazil	JX393993	JX394014	JQ968517	13
1C2011	*C. terrestris*	Pigeon dropping	Brazil	JX393994	JX394015	JQ968518	13
DS233	*C. terrestris*	Mopane tree	Botswana	KC469710	KC485476	KC469754	13
DS234	*C. terrestris*	Mopane tree	Botswana	KC469711	KC485477	KC469755	13
DS290	*C. terrestris*	Mopane tree	Botswana	KC469713	KC485479	KC469757	13
DS291	*C. terrestris*	Mopane tree	Botswana	KC469714	KC485480	KC469758	13
DS401	*C. terrestris*	Mopane tree	Botswana	KC469721	KC485487	KC469765	13
DS446	*C. terrestris*	Mopane tree	Botswana	KC469725	KC485491	KC469769	13
CBS 10810^T^	*C. terrestris*	Soil	USA	NA	EF370393	EU200782	NA
CBS 142^T^	*C. albidus*	Air	Japan	AB032616	AF075474	AF145321	14

NA: Not applicable.

T : Type strain. Hap: Haplotype number.

# : *C. aspenensis* sp. nov. (CBS 13867).

Mopane trees (*Colophospermum mopane*). Douglas fir (*Pseudotsuga menziesii*). Norway spruce (*Picea abies*). Trembling aspen (*Populus tremuloides*). CBS: Centraalbureau voor Schimmelcultures, Utrecht, The Netherlands. 18S-SSU: Small subunit rDNA. 28S-LSU: Large subunit rDNA. ITS: Internal transcribed spacer region.

All isolates were stored at −20°C in 70% YPD broth with 30% glycerol in 2-mL eppendorf tubes at the Mycology Laboratory at the Triangulo Mineiro Federal University (UFTM) for further analyses.

### DNA sequencing

Genomic DNA was extracted from yeast cells in accordance with previously described methodology [21]. The 5′ end of the 18S nuclear ribosomal small subunit rRNA gene (18S-SSU) (AFToL project available at http://aftol.org/primers.php), internal transcribed spacer (ITS) region [10], [22], and D1/D2 region of 28S-LSU [10], [23] were amplified from genomic DNA by PCR using the primers and conditions denoted in Table 2.

**Table 2:** PCR conditions and primers used for the amplification of the ribosomal loci.

Region	Forward	Reverse	Concentration	PCR Protocol
**18S-SSU**	NS-1: 5′-GTAGTCATATGCTTGTCTC-3′	NS-2: 5′-GGCTGCTGGCACCAGACTTGC-3′	50 pmol/each	94°C for 2 min; 36 cycles of 94°C for 1 min; 57°C for 1 min; 72°C for 2 min; 72°C for 15 min; and 4°C on hold
**28S-LSU**	NL-1: 5′-GCATATCAATAAGCGGAGGAAAAG-3′	NL-4: 5′-GGTCCGTGTTTCAAGACGG-3′	70 pmol/each	94°C for 2 min; 35 cycles of 94°C for 1 min, 57°C for 1 min; 72°C for 2 min; 72°C for 15 min; and 4°C on hold
**ITS**	ITS-1: 5′-GTCGTAACAAGGTTAACCTGCGG-3′	ITS-4: 5′-TCCTCCGCTTATTGATATGC-3′	70 pmol/each	94°C for 3 min; 29 cycles of 94°C for 30 s, 57°C for 30 s; 72°C for 45 s; 72°C for 10 min and 4°C on hold

PCR of the 18S-SSU, 28S-LSU, and ITS regions were performed using a PTC-100 Thermocycler (MJ Research Inc., Watertown, MA, USA) in a final volume of 50 µL. Each reaction contained 20 ng of genomic DNA, 1× PCR buffer (10 mmol L^−1^, Tris-HCl pH 8.3, 50 mmol L^−1^ KCl, and 1.5 mmol L^−1^ MgCl_2_), 0.25 mmol L^−1^ each of dATP, dCTP, dGTP and dTTP, and 1.25 U of *Taq* DNA polymerase (Invitrogen, São Paulo, SP, Brazil). The amplicons were stained with 0.5 mg mL^−1^ of ethidium bromide and visualized under UV light after two hours of electrophoresis at 80 V [22].

Each PCR product was independently sequenced with the forward and reverse primers of each region using the BigDye terminator v. 3.1 reagent kit (Applied Biosystems, Foster City, CA, USA) including *Taq* DNA polymerase (Invitrogen, São Paulo, SP, Brazil) in an automated DNA sequencer (ABI PRISM 3130×L Genetic Analyzer, Applied Biosystems, Foster City, CA, USA) according to the manufacturer's instructions.

### Sequencing analysis

Sequences were edited using the software Sequence Scanner V. 1.0 (Applied Biosystems, USA). Only nucleotide sequences with a Phred quality score ≥20 were included in our analysis to limit the possibility of incorporating an incorrect base to ≤1 in 100 (≥99% accuracy). Bioedit software was used to obtain consensus sequences from aligned forward and reverse sequence reads. Each consensus sequence was submitted to the Basic Local Alignment Search Tool (BLAST), and identity values ≥99% were obtained to assign species. All generated sequences were deposited in GenBank (Table 1) and The Barcode of Life Database (BOLD) (http://www.barcodinglife.org) [24].

### Phylogenetic relationships

Consensus sequences from newly identified isolates and those obtained from GenBank were aligned with the Clustal W2 algorithm (https://www.ebi.ac.uk/Tools/msa/clustalw2/) [25]. The phylogenetic analysis was calculated by the neighbor-joining, unweighted pair group method with arithmetic mean (UPGMA), and maximum likelihood methods in the MEGA 6.0 software [26]. For the neighbor-joining and maximum likelihood methods, the evolutionary distances were calculated in accordance with Kimura [27], while the Tamura 3-parameters method with the variation rate among sites modeled with a gamma distribution (shape parameter = 1) was used for UPGMA [28]. Phylogenetic relationships were calculated for each of the three regions and for the concatenated sequences applying a bootstrap analysis with 1,000 random resamplings. The type strain *Cryptococcus albidus* CBS 142 was designated as the outgroup in all phylogenetic analyses [29], [30]. Nucleotide sequences from the CBS-KNAW Fungal Biodiversity Centre (CBS) type strains were obtained from GenBank (Table 1).

To evaluate which of the three regions presented the highest variability, the intraspecific and interspecific pairwise distance was calculated by the Kimura 2-parameter model [27] in the MEGA 6.0 software [26].

### Haplotype network and goeBURST analysis

Haplotype networks were generated from the three concatenated sequence regions to visualize the differences and diversity among the *C. laurentii* isolates. The number and diversity of each haplotype were estimated using the software DNAsp v5.10 (http://www.ub.edu/dnasp/) [31]. Median-joining networks [32] for the concatenated dataset were obtained and graphed using the software Network 4.610 (http://fluxus-engineering.com).

To confirm the haplotypes obtained by median-joining networks the analyses were replicated in MLSTest software (available at http://mlstest.codeplex.com) and graphed by goeBURST algorithm in PHILOVIZ software [33], [34]. The minimum spanning tree representing the comparison between the isolates sources and their haplotype was also calculated by goeBURST.

### Coalescent species analyses

In order to estimate the time divergence between species and haplotypes, the interspecific and intraspecific net nucleotides substitutions (*d*) and standard error of the concatenated sequences were calculated in accordance to Kimura [27] with a bootstrap (500 replicates) as a variance method in the MEGA 6.0 software [35], [36]. The distance and standard error between closest species e. g. (*C. laurentii* x *C. rajasthanensis* 0.016±0.003; *C. aspenensis* x *C. flavescens* 0.071±0.007; *C. terrestris* x *C. flavescens* 0.009±0.002) were obtained and applied in the equation *d* = 2λ*t*, where *d* is the number of nucleotide substitutions per site between a pair of sequences, *t* is the divergence time, and λ the rate of nucleotide substitution. Here, we applied the constant (λ) 10^−9^/bp/year previously obtained for the *Eurotiomycetes* lineage due to the absence of a known fossil for *C. laurentii* species [36]. The resulting time of divergence were used as prior parameters to calibrate the tree in the coalescent analyses.

The optimal molecular evolutionary model for the concatenated sequences was determined using the corrected Akaike Information Criterion (AICc) as executed in the software jModelTest 2 [37], [38]. The optimal molecular evolutionary model General Time Reversible (GTR+I+G) with the respective parameters: AC: 0.7675, AG: 2.3377, AT: 1.8759, CG: 0.4999, GT: 1.0, alpha (IG): 0.5430, and pinv (IG): 0.5130 were obtained and used as priors in the coalescence analyses.

The BEAST software version 1.8.0 [39] was used to calculate the mean time to the most common recent ancestor (TMRCA) by the applying the Bayesian Markov-chain Monte Carlo (MCMC) method assuming a relaxed log-normal model of molecular rate heterogeneity. The chain lengths were 10^7^ generations with parameters sampled every 10^3^ generations with an initial burn-in off 10%. The posterior probability for a given clade was the frequency that the clade was present among the posterior trees which means that the probability of the lineage be considered monophyletic in the used dataset. Convergence of parameter values in the MCMC were assessed by the effective sample size (ESS) in the Tracer software version 1.6 [40].

### Nucleotide diversity of *C. laurentii* isolates

The extent of DNA polymorphisms, such as the number of polymorphic sites (S), nucleotide diversity (π), number of haplotypes (h), haplotype diversity (Hd), and average number of nucleotide differences (k), were calculated using DNAsp v5.10 [31]. In addition, Tajima's D, Fu & Li's D*, Fu & Li's F*, and Fu's Fs tests for neutrality were calculated. Negative or positive results in these tests suggest evidence of purifying or balancing selection, respectively.

### Fluorescence-activated cell-sorting (FACS) analysis

The FACS protocol was modified from Tanaka et. al. [41]. Cells were grown overnight at 25°C in YPD broth, collected by centrifugation, and washed with 1× PBS. Cells were then fixed in 1 ml of 70% ethanol overnight at 4°C with mild agitation. Cell pellets were obtained by centrifugation and the supernatants were discarded. Cells were resuspended and washed with 1 mL of NS buffer (10 mM Tris-HCl pH 7.2, 0.25 M sucrose, 1 mM EDTA, 1 mM MgCl_2_, 0.1 mM CaCl_2_, 0.55 mM Phenylmethylsulfonyl floride, 0.1 mM ZnCl_2_, 0.049% 2-mercaptoethanol). Cells were then resuspended in 180 µl NS buffer with, 14 mL RNase A (15 mg/µl, Qiagen) and 6 µl of Propidium iodide (1.0 µg/µl, CALBIOCHEM) and incubated in the dark for 4–8 hrs at room temperature. After incubating 50 µl of the cells were mixed with 500 mL of Tris-PI mix [482 µl 1M Tris pH 7.5+18 µl Propidium Iodide (1 µg/µl)]. Flow cytometry was performed on 10,000 cells with slow laser scan, on the FL1 channel with a Becton-Dickinson FACScan.

This study was approved by the Ethical Board of Triangulo Mineiro Federal University and is registered under the protocol number 32 CBIO/UFTM.

### Nomenclature

The electronic version of this article in Portable Document Format (PDF) will represent a published work according to the International Code of Nomenclature for algae, fungi, and plants, and hence the new names contained in the electronic version are effectively published under that Code from the electronic edition alone, so there is no longer any need to provide print copies. In addition, new names contained in this work have been submitted to MycoBank from where they will be made available to the Global Names Index. The unique MycoBank number can be resolved and the associated information viewed through any standard web browser by appending the MycoBank number contained in this publication to the prefix http://www.mycobank.org/MycoTaxo.aspx?Link=T&Rec=MB809723. The online version of this work is archived and available from the following digital repositories: PubMed Central and LOCKSS.

## Results

All isolates produced capsule and urease but not melanin and were phenotypically characterized as *C. laurentii* due to their ability to assimilate arabinose, α-methyl-D-glucoside, cellobiose, D-glucose, D-mannitol, D-ribose, D-trehalose, DL-lactate, dulcitol, galactose, inositol, L-rhamnose, lactose, maltose, melizitoze, raffinose, sacarose, sorbose, xylose, and 2-keto-glutarate. However, the isolates were negative for fermentation of dextrose and assimilation of inulin and potassium nitrate. FACS analysis indicated that most of the isolates are haploid (Figure S1).

**Figure 1:**
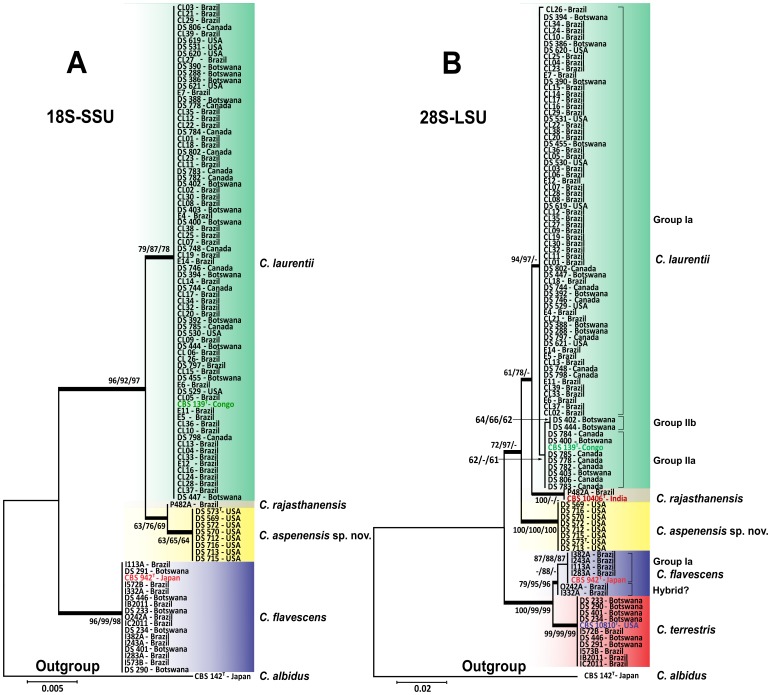
Phylogenetic analysis of 100 environmental *Cryptococcus* spp. isolates generated by the neighbor-joining, UPGMA, and maximum likelihood methods using partial nucleotide sequences of the (A) 5′end of 18S SSU-rDNA and (B) D1/D2 region of 28S LSU-rDNA. Numbers at each branch indicate bootstrap values>50% based on 1,000 replicates (NJ/UPGMA/ML). The analysis involved 103 and 105nucleotide sequences for the 18S-SSU and 28S-LSU respectively. ^T^: Type strain.

A 550-bp product was amplified from the 5′ end of 18S-SSU and sequenced with the primers NS-1 and NS-2, from which a 339-bp alignment was obtained. In this analysis, 75 isolates shared 99–100% identity with the *C. laurentii* CBS 139 (AB032640) type strain. Another 16 isolates shared 99% identity with *C. flavescens* CBS 942 (AB085796). The remaining 9 shared 99% identity with both *Bullera* sp. VY-68 (AB110694) from Japan and with *C. laurentii* RY1 from India (EF063147). High bootstrap values generated by neighbor-joining, UPGMA, and maximum likelihood analyses supported the differentiation of the following clades: *C. laurentii* (bootstrap values of 79, 87, and 78, respectively), *C. flavescens* (bootstrap values of 96, 99, and 98, respectively) and *Bullera* sp./*C. laurentii* (bootstrap values of 63, 65, and 64, respectively) (Figure 1A).

**Figure 2:**
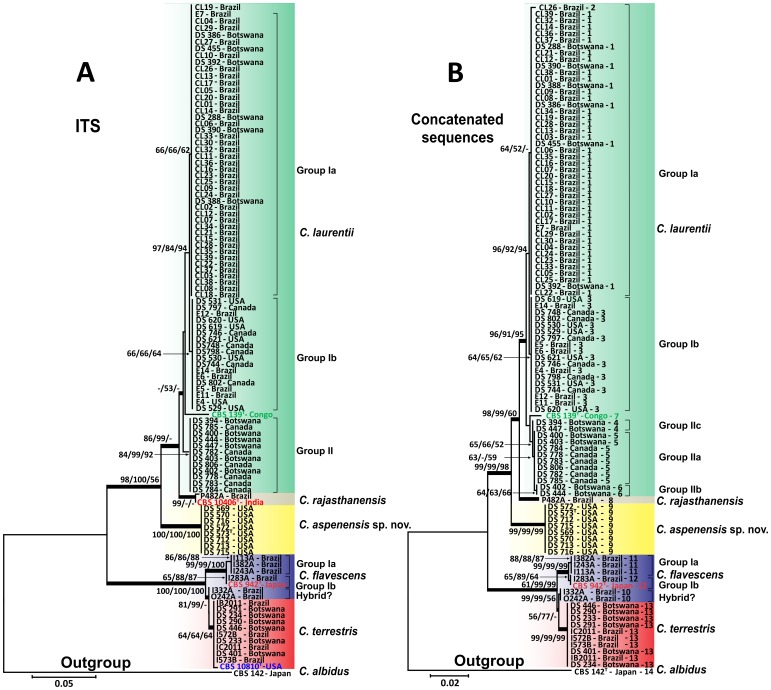
Phylogenetic analysis of 100 environmental *Cryptococcus* spp. isolates generated by the neighbor-joining, UPGMA, and maximum likelihood methods using partial nucleotide sequences of the (A) internal transcribed spacer (ITS) and (B) concatenated sequences of the three ribosomal regions. Numbers at each branch indicate bootstrap values>50% based on 1,000 replicates (NJ/UPGMA/ML). The analysis involved 105 and 103 nucleotide sequences for ITS and concatenated sequences respectively. ^T^: Type strain.

Due to the low genetic variability of the *C. laurentii* clade obtained at the 5′ end of the 18S-SSU gene, we sequenced two additional ribosomal loci: D1/D2 of the 28S-LSU and the ITS gene regions. The alignment and analysis of the 530-bp long amplicon of the 28S-LSU region confirmed the identification of 75 isolates as *C. laurentii* and showed more intraspecific variability differentiating three major groups (group Ia, IIa, and IIb) within *C. laurentii* isolates. Of the 16 *C. flavescens* isolates identified by the 18S-SSU sequencing, only 6 were confirmed in the *C. flavescens* clade by the 28S-LSU region with high bootstrap values of 79 (neighbor-joining), 95 (UPGMA), and 96 (maximum likelihood). The *C. flavescens* clade was split into two groups (Group Ia and a possible hybrid) by 28S-LSU and three groups (Groups Ia, Ib, and a possible hybrid) by ITS and analyses of the 1,328-bp amplicon of the concatenated regions. The two possible hybrid isolates I332A and O242A from Brazil were more related to *C. terrestris* in the ITS and concatenated sequences analyses (Figure 1B and 2). The remaining 10 isolates shared 99% identity with *C. terrestris* (CBS 10810), which has been recently described in Brazil and the United States (Figure 1B).

Among the nine *Bullera* sp./*C. laurentii* isolates identified by the 18S-SSU, isolate P482A shared 99% identity of the 28S-LSU and ITS regions with *Cryptococcus rajasthanensis* CBS 10406 (AM262324) from India. The eight remaining isolates (DS569, DS570, DS572, DS573, DS712, DS713, DS715, and DS716) recovered from a trembling aspen tree (*Populus tremuloides*) were designated as *Cryptococcus aspenensis* sp. nov. because they shared 100% identity with two undescribed isolates of *Cryptococcus* sp. APSS-862 (FM178286) and *Cryptococcus* sp. APSS-823 (AM931019) from India. These eight isolates exhibited a genetic distance of 3.8% and 7.1% from *C. rajasthanensis* and 2.3–2.7% and 6.4–7.3% from *C. laurentii* by 28S-LSU and ITS region analysis, respectively (Figure 1B and 2A).

**Figure 3:**
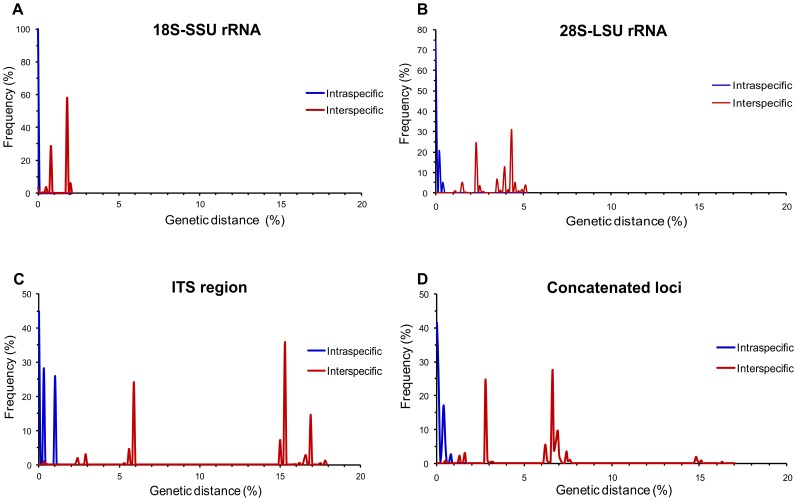
Intraspecific and interspecific pairwise distance of the three ribosomal regions of the environmental *Cryptococcus* spp. calculated by the Kimura 2-parameter model revealed higher variability of the ITS region compared with the 18S-SSU and 28S-LSU regions.

Overall, the pairwise distance of the three sequenced regions showed the highest intraspecific and interspecific variability in the ITS region (genetic distance higher than 15%) when compared with the 2.5% and 5.0% obtained with 18S-SSU and 28S-LSU, respectively (Figure 3).

**Figure 4:**
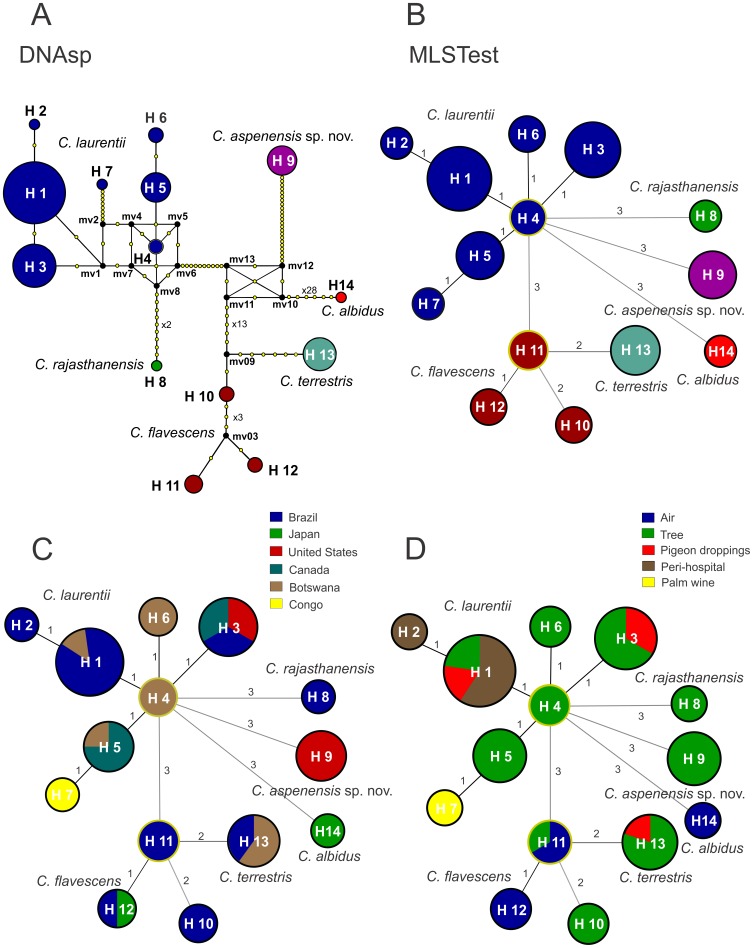
Median-joining haplotype network (A) of environmental *C. laurentii* isolates based on concatenated nucleotide sequences of the 5′ end of 18S-SSU, D1/D2 of 28S-LSU, and ITS regions. The tree represents 103 *Cryptococcus* spp. isolates from Brazil, Botswana, Canada, Japan, India, and the United States. The seven *C. laurentii* and three *C. flavescens* haplotypes are clearly distinguished. The Botswana ancestral haplotype (H4) of *C. laurentii* is presented and highlighted in yellow. Each circle represents a unique haplotype (H), and the circumference is proportional to haplotype frequency (H1: 44 isolates; H2: 1; H3: 18; H4: 2; H5: 8; H6: 2; H7: 1; H8: 1; H9: 8; H10: 2; H11: 3; H12: 2; H13: 10; H14: 1; outgroup *C. albidus* CBS 142). Yellow dots represents the number of mutation sites, excluding gaps, between the haplotypes. Black dots (median vectors) are hypothetical missing intermediates. Minimum spanning trees (B) using the goeBURST algorithm confirm the haplotype relationships among *C. laurentii* isolates determined by median-joining network analysis. The size of the circle corresponds to the number of isolates within that haplotype, and the numbers between haplotypes represent the genetic distance of each haplotype, excluding the gaps. Minimum spanning trees as described in B modified to show the distribution of haplotypes according to the country of origin (C) or environmental source (D).

The haplotype diversity of the concatenated regions was assessed using DNAsp and MLSTest software. Multiple haplotype groups were identified within *C. laurentii* and *C. flavescens*, but not the *C. aspenensis* sp. nov. and *C. terrestris* (Figure 4A and 4B). The *C. laurentii* isolates were represented by seven haplotypes (H1 to H7). Haplotype 1 (H1) included 44 isolates, of which 38 (86.4%) were from Brazil, followed by the H3 composed of 6 from Brazil, 6 isolates from Canada, and 6 from the United States (Figure 4C and 4D). The highest genetic distance (12 polymorphisms) in the *C. laurentii* haplotypes was observed between H7 (CBS 132 type strain) and H6 (DS402 and DS444 isolates). Five of the seven *C. laurentii* isolates were recovered from Africa despite very limited sampling; three were unique haplotypes (H4, H6, and H7) and two were only observed in Brazil (H1) or Canada (H5). H4, which was obtained from Mopane trees in Botswana, was identified as the ancestral of *C. laurentii* in both Network and MLSTest analyses. H7 (*C. laurentii* type strain CBS 139) was restricted to the Congo and was in much closer proximity to Botswana than any other sample region. H3 was distinct from Botswana and was comprised of isolates from North and South America (Figure 4).

Three haplotypes were identified in *C. flavescens* isolates (H10, H11, and H12), with the ancestral haplotype H11 restricted to Brazil. H10 presented the highest genetic distance (9 polymorphisms) when compared with H11 and H12 (2 polymorphisms). H10 was also positioned closer to the *C. terrestris* haplotype H13 and could be a unique species, or ancestral genotype, or recombinant hybrid isolate between *C. flavescens* and *C. terrestris*. The *C. aspenensis* sp. nov. H9 was a completely unique genotype from New York, USA (Figure 4).

**Figure 5:**
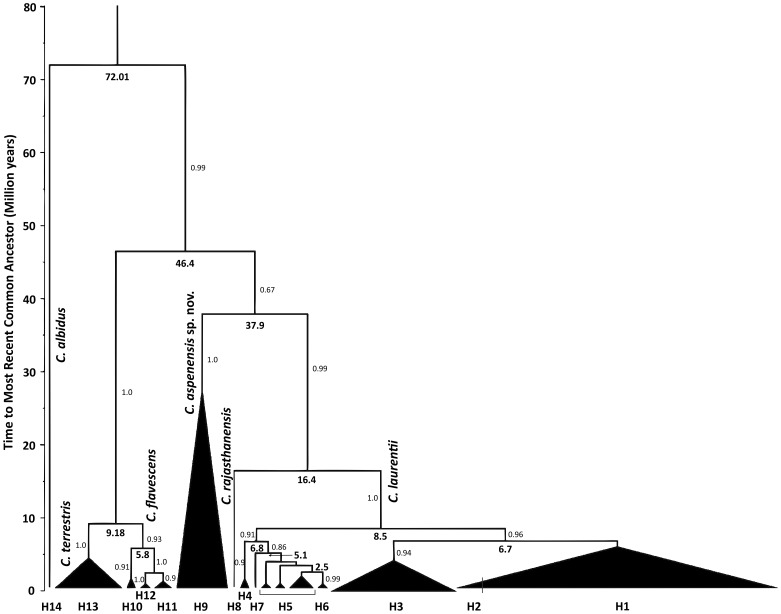
Species tree of the *C. laurentii* species complex resulting from coalescent analyses of the concatenated data set. The speciation of *C. aspenensis* from *C. laurentii* and *C. rajasthanensis* took place 37.9 million years ago. The *C. laurentii* haplotype (H4) from Botswana was the first haplotype to be differentiated (6.8 million years ago). Numbers at branches represent the Bayesian posterior support values while the bold numbers represent the nodes ages (in millions of years).

Estimates of the mean time to divergence for the *C. flavescens* and *C. terrestris* isolates were 4.02–5.4±0.87 million years (about 9 million years ago) with an effective sample size (ESS) of 1213.3 and 1006.8, respectively. For *C. laurentii* population, the TMRCA were 8.03±1.83 million years (about 16.4 million years ago) (ESS = 6615.0) while for the new species *C. aspenensis* sp. nov. 26.7±3.9 million years (about 37.9 million years ago) (ESS = 355.5). Coalescent analysis was strongly supported with (>95.0) Bayesian posterior values (Figure 5). Phylogenetic and coalescent analyses agree demonstrating additional support for the recognition of additional related haplotypes and species.

The *C. laurentii* nucleotide sequences of the 18S-SSU, 28S-LSU, ITS, and the concatenated regions presented 0, 3, 11, and 14 polymorphic sites, respectively (Table 3). The highest nucleotide diversity (π) of 0.0039 was observed for ITS. Low values of haplotype (Hd = 0.604) and nucleotide diversity (π = 0.0014) of the concatenated regions may suggest clonal reproduction in this species (Table 3).

**Table 3:** DNA polymorphisms in the ribosomal loci of the 75 *C. laurentii* environmental isolates.

Region	Length	*S*	*π*	*k*	*h*	*H_d_*	*D*	*F_D_*	*F_F_*	*F_S_*
**18S- SSU**	399	0	0.0	0.0	1	0.0	-	-	-	-
**28S- LSU**	530	3	0.0006	0.3291	4	0.280	−0.873	−0.521	−0.737	−1.537
**ITS**	399	11	0.0039	1.5881	4	0.570	−0.977	−2.674^a^	−2.472^a^	2.852
**Concatenated**	1328	14	0.0014	1.9112	7	0.604	−1.079	−2.502^a^	−2.373^a^	0.731

***S***: number of polymorphic sites. ***π***: nucleotide diversity. ***k***: average number of nucleotide differences per sequence. ***h***: number of haplotypes. ***H_d_***: haplotype diversity. ***D*** **,** ***F_D_*** **,** ***F_F_*** **and** ***Fs***: Tajima's D, Fu and Li's D*, Fu and Li's F* and Fu's Fs, respectively.

a : p value<0.05.

### Taxonomy

***Cryptococcus aspenensis***. Ferreira-Paim, K., Ferreira, T. B., Andrade-Silva, L., Mora, D. J., Springer, D. J., Heitman, J., Fonseca, F. M., Matos, D., Melhem, M. S. C., et Silva-Vergara, M. L. sp. nov. [urn:lsid:mycobank.org:names:MB809723].

**Figure 6:**
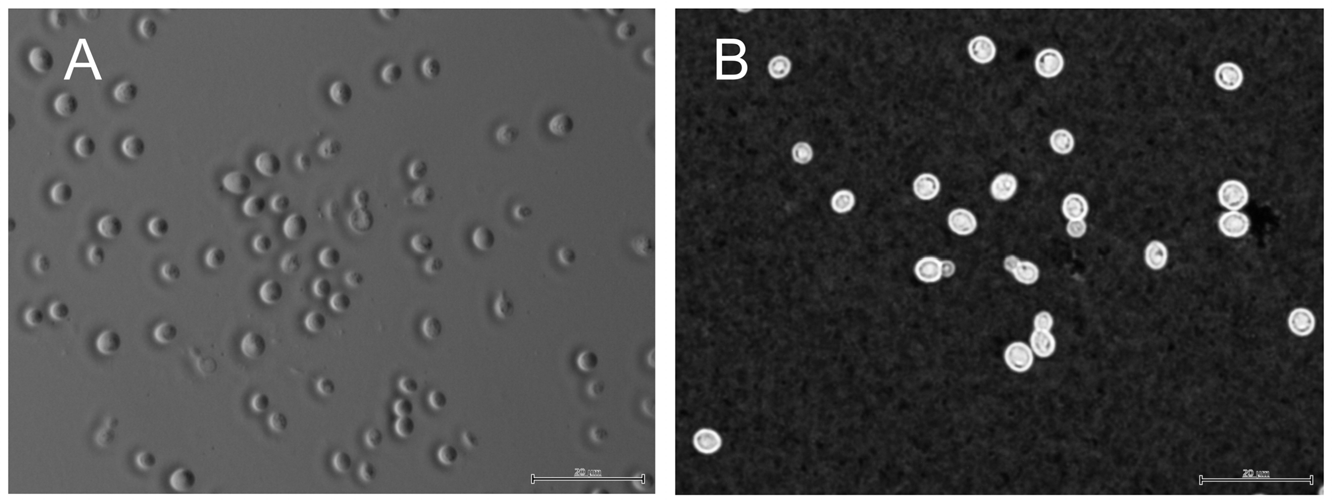
Differential interference contrast (A) and India Ink staining (B) of *C. aspenensis* sp. nov. DS573^T^ (CBS 13867) cells after 3 days at 25°C in YPD broth. Scale bar of 20 µm is shown.

After 3 days on YPD agar at 25°C, *Cryptococcus aspenensis* colonies are circular, cream-colored with an entire margin, smooth, mucous to butyrous, glistening, and raised. Growth (poor) at 37°C was also observed. After 3 days at 25°C in YPD broth, the cells are ellipsoid to globose (7.5–8.7 to 5–6.2 µm), and they may be single or with one attached polar bud (Figure 6). After 15 days in slide cultures on cornmeal agar, pseudomycelium or mycelium is not formed. Fermentation ability is negative. Arabinose, α-methyl-D-glucoside, cellobiose, D-glucose, D-mannitol, D-ribose, D-trehalose, DL-lactate, dulcitol, galactose, inositol, L-rhamnose, lactose, maltose, melizitoze, raffinose, sacarose, sorbose, xylose, and 2-keto-glutarate are assimilated. Cells were haploid by FACS analysis (Figure S1).

Unambiguous identification and phylogenetic placement is based on DNA sequences of the following nuclear loci: ITS (KC469778), 18S-SSU (KC469734), D1/D2 of 28S-LSU (KC485500). The type strain DS573 was isolated from the bark of a trembling aspen (*Populus tremuloides*) in the New York, United States and has been deposited in the Centraalbureau voor Schimmelcultures, The Netherlands, as CBS 13867 and in the Westmead Millennium Institute, Australia, as WM 14.137. Other strains belonging to this species include DS570 (CBS 13868, WM 14.138), and DS715 (CBS 13869, WM 14.139) which were isolated from a single trembling aspen tree in New York.

**Etymology**: The specific epithet *aspenensis* L. adj. *aspenensis* associated with trembling aspen (*Populus tremuloides*), the tree substrate from which the type strain was isolated.

## Discussion

Fungal identification and taxonomy has markedly improved during the last decade and as a result, several recognized species, such as *Sporothrix schenckii*, *Paracoccidioides brasiliensis*, and *Coprinopsis cinerea*, have been distinguished as cryptic species complexes [35], [42], [43]. In this context, the sequencing of the 18S-SSU, D1/D2 of 28S-LSU, and ITS of the ribosomal region have been useful in yeast identification for more than 10 years. However, the low variability of the 18S-SSU and 28S-LSU regions may prohibit identification of cryptic species, while the variability of the ITS region has been frequently utilized for fungal phylogenetic studies and the fungal tree of life barcoding projects (http://tolweb.org) [44], [45].

*C. laurentii* has classically been considered a saprophytic yeast, although 24 cases of human infection have been described, suggesting that *C. laurentii* is an opportunistic pathogen with potential similarities to the distantly related pathogenic *C. neoformans* and *C. gattii* species [3], [44], [46]–[48]. Cryptococcosis due to *C. laurentii* has been associated with severely immunocompromised patients and/or those presenting with other underlying diseases. In such cases, *C. laurentii* was most frequently isolated from the blood, but also from several other body sites such as the CSF, skin, and lungs [49], [50].

In this study, we evaluated 100 phenotypically identified *C. laurentii* isolates from several countries. Of these, 75 were confirmed to be *C. laurentii* by phylogenetic analysis of the 18S-SSU, 28S-LSU, and ITS regions. The obtained sequences shared 99–100% identity with sequences from Brazil, China, South Africa, and the United States, demonstrating its worldwide and overlapping geographic distribution with *C. neoformans* and *C. gattii*. Although, in North America, *C. gattii* has been associated with clinical infection in patients from New York, Rode Island, and other states [51]–[53]. At present, *C. gattii* has only been environmentally isolated from the Western United States [54] and Canada [55], while *C. neoformans* is broadly associated with pigeon guano in many regions of the United States, including the state of New York [56]. Hence, our study suggests that in the United States, *C. laurentii* appears have a much broader distribution than *C. gattii* as noted from its isolation in association with grasses in the USA, and goose guano and trees in New York State [57].

Within the *C. laurentii* clade, intraspecific variability of 0.2% (1 polymorphism), 0.2–0.4% (1–3 polymorphisms), and 0.3–2.4% (1–11 polymorphisms) was obtained for the analysis of the 18S-SSU, 28S-LSU, and ITS regions, respectively. These features are consistent with a previously published report indicating that one polymorphism exhibited in the 28S-LSU region exist up to 11 substitutions in the ITS region [58]. Through phylogenetic analysis of the 28S-LSU and ITS regions, three divergent groups were distinguishable from the 75 *C. laurentii* isolates. Groups IIa and II of the 28S-LSU and ITS regions contained eight isolates from Botswana and Vancouver, which differed from the remaining 67 isolates in 1–3 and 5–11 nucleotides in the 28S-LSU and ITS regions, respectively, and constituted H5 in the network and goeBURST analysis. Additional analysis of environmental and clinical samples from outside of Brazil will be valuable to determine whether H5 is distinct to Brazil. The majority of Brazilian isolates are H1 (44 isolates). The high frequency of the H1 haplotype may be related to microevolution and/or adaptation of these isolates to the environment, while the H2 haplotype may be rare.

Despite the differences in the total number of *C. laurentii* isolates, those from Botswana (n = 12) shared five of the seven haplotypes observed, two of them unique (H4 and H6). Interestingly, the ancestral H4 is only represented in Botswana suggesting that similar to *C. neoformans* var. *grubii*, *C. laurentii* may have originated from Africa [59]. The historical haplotype (H7) from palm wine is also restricted to the Congo, which is near to Botswana. Other haplotypes common in Africa are only also observed from Brazil (H1) or Canada (H5). Therefore, it is possible that *C. laurentii* was introduced into Brazil and Canada from Africa. To test this hypothesis, the coalescent gene analyses was performed which showed that the isolates within the haplotype 4 are the oldest in our data set (6.8 million years ago).

The remaining 25 isolates that were originally identified as *C. laurentii* by standard phenotypic assays were identified by ITS, 18S-SSU, and 28S-LSU analyses as *C. terrestris* (n = 10), the *C. aspenensis* sp. nov. (n = 8), *C. flavescens* (n = 6), and *C. rajasthanensis* (n = 1). *C. rajasthanensis* isolates are rare, and few have been previously reported in GenBank from India, Thailand, China, and Brazil. The *C. rajasthanensis* isolate in our study was recovered from hollow trees in São Paulo, Brazil and differed from *C. laurentii* by 0.4–0.6%, 1.7–2.1%, and 4.3–4.8% in the 18S-SSU, 28S-LSU, and ITS regions, respectively. In previous studies, the *C. laurentii* type strain (CBS 139) differed from the known Indian *C. rajasthanensis* reference isolate (CBS 10406) by 1.6% in the 28S-LSU region and 7.5% in the ITS region.

Despite the genetic distance observed between *C. flavescens* and *C. laurentii* (4–5.2% in 28S-LSU and 16.8–18.9% in the ITS), the species have long been considered phenotypically indistinguishable. For example, one previously identified clinical isolate of *C. laurentii* was later distinguished to be *C. flavescens* [4], [60], suggesting that opportunistic pathogen traits may have evolved more than once within this group, similar to the presence of sporadic opportunistic pathogens in *Kwoniella* and *Cryptococcus heveanensis* species groups [61].

*C. flavescens* has only recently been differentiated as a sibling species of *C. terrestris* [13] with the advancements in multi-locus sequence analysis. It is likely that the delayed recognition of *C. terrestris* and *C. flavescens* hindered the recognition of divergent phenotypic traits now recognized as important species characteristics. *C. terrestris* can be differentiated phenotypically from *C. flavescens* by delayed and/or weak assimilation of ribose and salicin [13], [44]. Our analysis supports the previous reported genetic differentiation; *C. flavescens* diverged from *C. terrestris* by 1.2–1.6% (6–10 polymorphisms) and 0.5–2.5% (2–10 polymorphisms) in the 28S-LSU and ITS regions, respectively. This difference probably occurred 9.1 million years ago as demonstrated by the coalescent analyses.

The six *C. flavescens* isolates recovered from Brazil were similar to isolates from China, Egypt, Italy, Japan, South Africa, and the United States, confirming the ubiquity of this species. The intraspecific variability of 0.2%, 0.4% and 0.8–2.2% observed in the 18S-SSU, 28S-LSU, and ITS regions, respectively, and the description of one haplotype in 18S-SSU, two in 28S-LSU, and three in the ITS region and concatenated analyses for the first time is relevant in the biological context of this species. Both isolates within H10 (I332A and O242A) share higher similarity with *C. terrestris* in 18S-SSU, the ITS region, and concatenated sequence but are more similar to *C. flavescens* in 28S-LSU. H10 may be a second haplotype of *C. terrestris* or a possible hybrid haplotype between the two species, as has been observed between *C. gattii* and *C. neoformans* [62]–[64]. Both isolates within this unique haplotype appear haploid by FACS which suggest this isolate may be a recombinant between *C. flavescens* and *C. terrestris* or a ancestral genotype. Coalescent analysis does not support the hypothesis that the two isolates in haplotype 10 are ancestral to both species and hence it is likely this haplotype arose from a productive introgression between *C. flavescens* and *C. terrestris*. Whole genome sequencing and the development of multilocus sequence primers specific to *C. laurentii* will be needed to support these hypotheses. Furthermore, the ancestral haplotype of *C. flavescens* appears to be H11 (revealed by MLSTest but not by DNAsp), which is confined to Brazil, suggesting that it may have originated in Brazil. Additional environmental and clinical isolates must be evaluated to better define the place of origin of *C. flavescens*.

A newly identified but distinct haploid group that we designated as *C. aspenensis* sp. nov. was identified consistently through phylogenetic analyses of individual and concatenated loci and confirmed by coalescent analyses. At present, this constitutes a previously unidentified species that appears to be restricted to New York, United States. All eight isolates obtained in the H9 group appear to be nearly identical/clonal and were obtained from sampling one trembling aspen tree in Long Island, New York, United States. An additional isolate was just identified from soil sample collect on July 13 in Copake, New York (personal communication D. J. Springer) and supports the recognition of this newly identified species. *C. aspenensis* sp. nov. appears to represent a unique ancestral lineage that diverged from the common ancestor prior to *C. rajasthanensis* and *C. laurentii* approximately 37 million years ago.

With the advent of inexpensive sequencing, alignment, and analysis, increasing numbers of sequences for bacteria, plants, viruses, animals, protozoa, and fungi are rapidly being deposited in publically accessible databases such as GenBank [65]–[67]. In fungi, several regions have been utilized for phylogenetic studies including the ITS, 28S-LSU, and 18S-SSU of the rRNA cistron regions, as well as *CO1* (*Penicillium*), MCM7 (ascomycetes), and RBP1 (Assembling the Fungal Tree of Life, AFToL project) [45], [58], [66]. Schoch et al. recently reported that the ITS region was generally superior to the LSU in species discrimination and had a more clearly defined barcode gap, indicating that the ITS region should be designated as the universal barcode for fungi [45]. Our analyses concur with this previously published report; we found increased variability in the ITS region that resulted in better phylogenic differentiation between the highly related, globally distributed, and potentially clonal *C. laurentii* species group. Concatenated sequence analysis resulted in the identification of novel and distinct haplotypes within *C. laurentii* that appear to be associated with specific geographic regions.

Additional analysis of clinical and environmental specimens, mating type determination, sequencing of housekeeping genes, and whole genome analysis are required to further resolve potential haplotypes within *C. laurentii* and resolve the phylogenetic placement of the closely related species *C. rajasthanensis*, *C. flavescens*, *C. terrestris*, and the *C. aspenensis* sp. nov. described in this analysis.

## Supporting Information

Figure S1 **Representative Fluorescence-activated cell-sorting (FACS) analysis of the** ***Cryptococcus*** **spp. included in the study.** All isolates except three *C. laurentii* (CL11, CL19, and E11) and one *C. flavescens* (I234A) appear haploid. Positive haploid (CBS10574) and diploid controls (XL143) were included in each FACS run.
(TIF)
