# Supplementary figures and images for: Phylogenetic Analysis of Phenotypically Characterized Cryptococcus laurentii Isolates Reveals High Frequency of Cryptic Species
Source: PLoS One. 2014 Sep 24;9(9):e108633. doi: 10.1371/journal.pone.0108633 (PMC4177401; doi:10.1371/journal.pone.0108633)

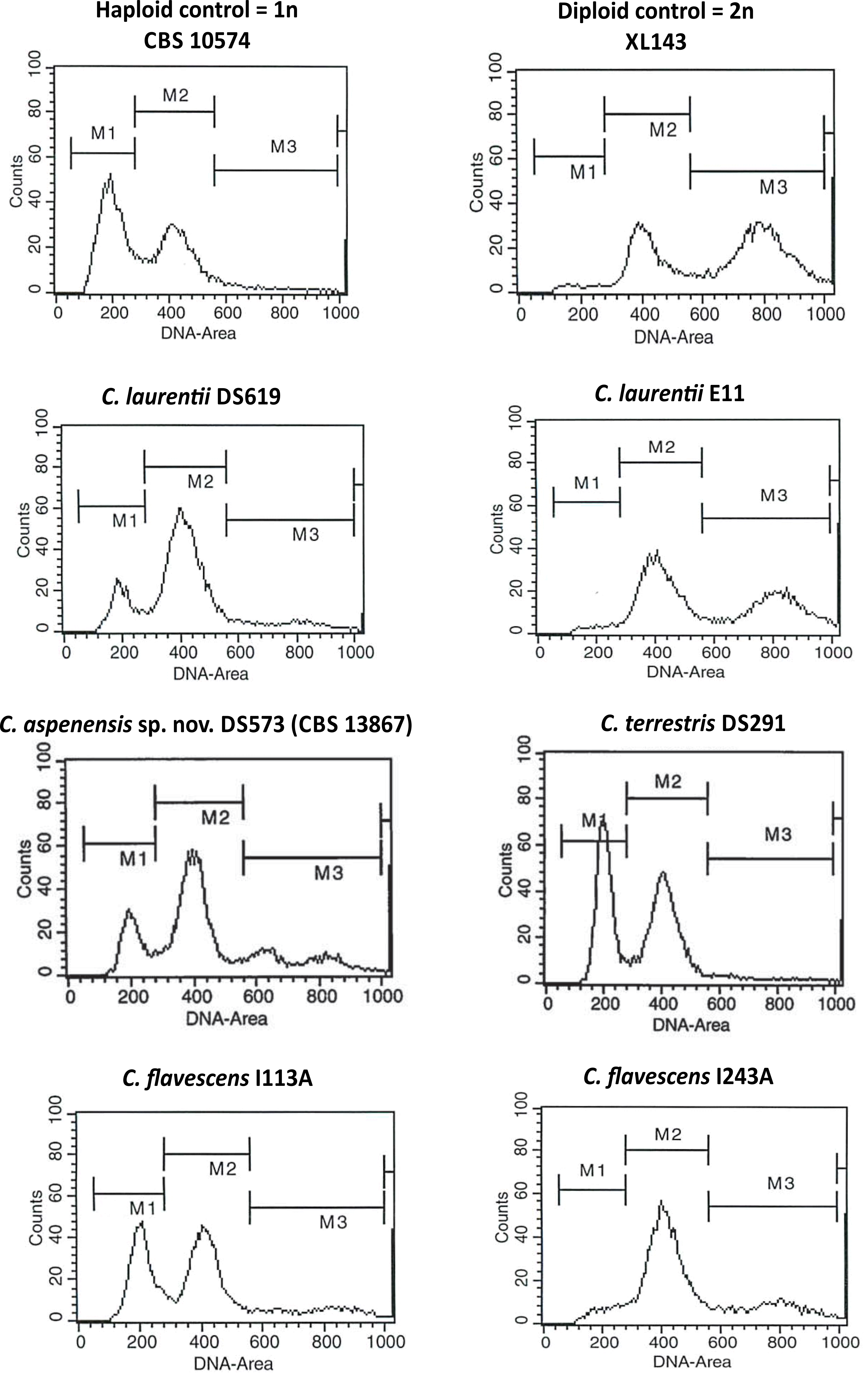

Supplement: Figure S1 — Representative Fluorescence-activated cell-sorting (FACS) analysis of the Cryptococcus spp. included in the study. All isolates except three C. laurentii (CL11, CL19, and E11) and one C. flavescens (I234A) appear haploid. Positive haploid (CBS10574) and diploid controls (XL143) were included in each FACS run. (TIF) [file pone.0108633.s001.tif]
